# REM density predicts rapid antidepressant response to ketamine in individuals with treatment-resistant depression

**DOI:** 10.1038/s41386-025-02066-7

**Published:** 2025-02-15

**Authors:** Mina Kheirkhah, Wallace C. Duncan, Qiaoping Yuan, Philip R. Wang, Hamidreza Jamalabadi, Lutz Leistritz, Martin Walter, David Goldman, Carlos A. Zarate, Nadia S. Hejazi

**Affiliations:** 1 Experimental Therapeutics and Pathophysiology Branch, Intramural Research Program, National Institute of Mental Health, National Institutes of Health, Bethesda, MD USA; 2 Department of Psychiatry and Psychotherapy, Jena University Hospital, Jena, Germany; 3 National Institute on Alcohol Abuse and Alcoholism, National Institutes of Health, Bethesda, MD USA; 4 Department of Psychiatry, Brigham and Women’s Hospital, Boston, MA USA; 5 Department of Psychiatry and Psychotherapy, Philipps University of Marburg, Marburg, Germany; 6 Institute of Medical Statistics, Computer and Data Sciences, Jena University Hospital, Jena, Germany

**Keywords:** REM sleep, Depression

## Abstract

Abnormalities during rapid eye movement (REM) sleep contribute to the pathophysiology of major depressive disorder (MDD), but few studies have explored the relationship between REM sleep and treatment-resistant depression (TRD). In MDD, REM sleep abnormalities often manifest as alterations in total night REM Density (RD), RD in the first REM period (RD1), and REM Latency (RL). Among these, RD1 is notably considered a potential endophenotype of depression. This study compared REM sleep markers between 63 drug-free individuals with TRD (39 F/24 M) and 41 healthy volunteers (25 F/16 M). It also investigated the effects of ketamine, an N-methyl-D-aspartate (NMDA) receptor antagonist, on these REM sleep variables. Specifically, the study investigated whether RD1 could predict antidepressant response to ketamine. TRD participants showed higher RD1 and shorter RL at baseline compared to HVs, as assessed via non-parametric tests, but Total Night RD did not differ between the two groups. Ketamine treatment decreased RD1 in TRD participants but did not affect Total Night RD or RL. As assessed via the Support Vector Machine (SVM) algorithm, baseline RD1 level moderately predicted antidepressant response to ketamine versus non-response (area under the receiver operating characteristic (ROC) curve (AUC) = 0.73, with a median accuracy of 0.75), wherein TRD participants with higher baseline RD1 were more likely to respond to ketamine. These results underscore the utility of RD1 for identifying individuals most likely to benefit from ketamine treatment, enabling more targeted and effective therapeutic strategies. Clinical Trials Identifier: NCT00088699, NCT01204918.

## Introduction

Abnormalities in rapid eye movement (REM) sleep are well-documented in major depressive disorder (MDD) [1–4]. Increased REM Density (RD), particularly during the first REM period (RD1), is present in individuals with MDD and their first-degree relatives [5–8]. RD1 is considered an endophenotype for depression [9–11], persisting during remission and indicating lasting vulnerability [5–8]. Previous studies have also linked higher baseline RD to better treatment outcomes in depression. For instance, higher RD at baseline predicted favorable response to selective serotonin reuptake inhibitors (SSRIs) and selective norepinephrine reuptake inhibitors (SNRIs) [12], and Göder and colleagues reported that higher RD1 at baseline also predicted better response to electroconvulsive therapy (ECT) [13]. The same study also found that ECT reduced RD1 levels, which suggests that RD and RD1 are not rigid trait markers. Another variable of interest is REM Latency (RL). Shortened RL has been linked with decreased sleep continuity and serves as biological marker for vulnerability to depression [14] and predicting relapse [5, 8, 15, 16].

Despite these findings, few studies have explored the relationship between REM sleep and treatment-resistant depression (TRD), in which symptoms persist despite multiple treatment interventions. In the US, TRD affects about 30% of those diagnosed with MDD [17]. Interestingly, nearly a third of individuals with TRD have a rapid antidepressant response to the N-methyl-D-aspartate antagonist ketamine [18]; its effects on sleep include increased delta power earlier in the night and an overall enhancement of slow wave sleep (SWS) [19–23]. However, little is known about ketamine’s impact on REM sleep variables or about the relationship between REM sleep variables such as RD and RD1 and antidepressant response to ketamine in individuals with TRD. Given the association between RL [10, 24, 25], RD [4, 26, 27], and depressive states and traits [8, 9], further investigation of REM in the context of TRD seems warranted.

This study compared Total Night RD, RD1, and RL between medication-free TRD participants and healthy volunteers (HVs) at baseline and evaluated ketamine’s effects on these variables. The study hypothesis was that ketamine would increase RL and decrease both Total Night RD and RD1. The study also sought to determine whether RD1 could predict antidepressant response to ketamine in individuals with TRD. As a secondary objective, the study also assessed ketamine’s effects on additional nighttime sleep variables, including total sleep time (TST), REM Efficiency, REM Time, SWS, and wake after sleep onset (WASO).

## Materials and methods

### Participants

Unmedicated adults with TRD (*n* = 63 (39 F/24 M), 20–65 years old, mean age = 40.76 ± 11.57 years) and HVs (*n* = 41 (25 F/16 M), 20–57 years old, mean age = 34.49 ± 10.66 years) participated in a series of inpatient, randomized, controlled trials conducted at the National Institutes of Health (NIH) Clinical Center in Bethesda, MD between 2006 and 2018 (NCT00088699, NCT01204918); results have previously been published [28–30]. The TRD group included both individuals with bipolar disorder (*n* = 27) and MDD (*n* = 36). This study was a secondary analysis of individuals who participated in two trials within a larger protocol, “The Investigation of the Rapid (Next Day) Antidepressant Effects of an NMDA Antagonist” (NIH protocol 04-M-0222), and the analyses presented here are exploratory. Participants were required to discontinue psychotropic medications for at least two weeks prior to the study (five weeks for fluoxetine and three weeks for aripiprazole). Participants stayed in the unit during the washout period and were closely monitored by nursing staff and attendants to ensure adherence to the protocol.

Baseline sleep data for all participants were collected the night before a single ketamine infusion (0.5 mg/kg IV). Post-ketamine sleep data were collected on the day of ketamine infusion (see Polysomnography section, below, for additional details). A CONSORT diagram is provided in Supplementary Figure S1.

TRD inclusion criteria for the present analysis included: 1) a diagnosis of MDD without psychotic features, confirmed using the Structured Clinical Interview for Axis I DSM-IV Disorders (SCID)-Patient version [31], administered by a trained clinician; 2) a Hamilton Rating Scale for Depression (HAMD-17) [32] score ≥ 17 at baseline (the mean baseline HAM-D score for the TRD group was 21.45 ± 3.98); 3) a history of non-response to one or two FDA-approved antidepressants administered at an adequate dose and duration, in accordance with the definition of TRD used during the original clinical trials. In this context, a trial of ECT counted as an adequate antidepressant trial; and 4) a current depressive episode lasting at least four weeks. Exclusion criteria included a DSM-IV diagnosis of drug or alcohol dependence or abuse in the last three months or serious or unstable medical illness. Participants with diagnosed obstructive sleep apnea (OSA) or those recently screened for apnea at external facilities were excluded from the study, and the STOP-BANG questionnaire [33] and Stanford Sleepiness Scale (SSS) [34] were also used to identify participants at high risk for OSA, particularly males and older adults with comorbidities. Anxiety disorders were not exclusionary.

The HAM-D was used as the primary measure to define response to ketamine because, unlike the Montgomery-Asberg Depression Rating Scale (MADRS), it includes items specifically designed to assess physiological and sleep-related symptoms, which were closely aligned with the study’s focus on sleep variables and disturbances [35, 36] and made the HAM-D more suitable for testing our specific hypothesis [12, 13]. Additional analyses with the MADRS were conducted to test this hypothesis, and no significant baseline differences in RD1 levels were observed between responders and non-responders (see Supplementary Figures S2 and S3). Antidepressant response to ketamine was defined as achieving a 50% reduction in HAM-D score one day after administration. Participants who met this criterion were classified as responders (n = 20), and those who did not were considered non-responders (*n* = 41) to ketamine. Two of the 63 TRD participants were excluded because data about their response to ketamine were missing.

Eligibility criteria for HVs included age between 18–65 years, absence of Axis I disorders according to the SCID Non-Patient version [37], and absence of first-degree relatives with DSM-IV Axis I disorders.

The clinical trials from which these data were drawn (NCT00088699 and NCT01204918) was approved by the Combined Neuroscience Institutional Review Board of the NIH. Participants provided written informed consent before entry into their respective studies and were assigned a clinical research advocate to independently monitor the consent process and ethical research participation.

### Polysomnography (PSG)

**Fig. 1:**
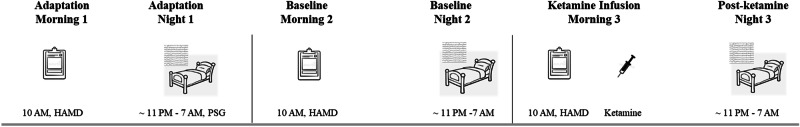
Timeline of sleep study nights. Hamilton Depression Rating Scale (HAM-D) ratings, and ketamine infusion.

Both TRD and HV participants completed three consecutive nights of sleep assessment using laboratory-based polysomnography (PSG) conducted by trained sleep technicians. Night 1 served as an adaption night, allowing participants to acclimate to the sleep lab environment. Night 2 served as the baseline sleep study, and Night 3 was conducted to assess the effects of ketamine. Ketamine was administered at approximately 10:30 am, and the post-treatment sleep study was conducted later that night around 11:00 pm. Figure 1 provides a detailed timeline of the sleep study nights, including the timing of HAM-D ratings and ketamine infusion. Participants selected a Lights-Out time around 11:00 pm, aligning with their regular sleep schedule; the latest Lights-On time was set at 7:00 am. As per the protocol, participants were encouraged to avoid napping and were specifically prohibited from napping for three days before and after their sleep study session. Electroencephalograms (EEGs) were recorded from electrode sites F3-A2, F4-A1, C3-A2, C4-A1, O2-A1, and O1-A1. In addition, left and right electrooculograms and submental electromyograms were collected using a Nihon Kohden system (Neurofax Sleep v. 05–50; Nihon Kohden Corporation, Japan) and Polysmith Acquisition and Review software (v. 4.0 and v. 10.0; Nihon Kohden) with a sampling rate of 200 Hz. EEG recordings were visually scored by two independent, blinded reviewers in 30-second epochs, following established criteria [27].

### Total night RD, RD1, and RL measures

Total Night RD was calculated as the total number of eye movements during the entire night divided by the total duration of REM Time across the night. RD1 was calculated as the number of eye movements per minute during the first REM period (REMP) divided by the total REM Time in that period. Rapid eye movements were identified by voltage changes > 25 µV [27, 38] without a duration criterion. RL time was defined as the time between sleep onset and the first REMP. A REMP was defined as lasting at least three minutes, based on scoring guidelines established by Rechtschaffen and Kales [39]; distinct REMPs were separated by 15 min or more of non-REM sleep [40].

### Additional nighttime sleep measures

Sleep onset was defined as the first epoch of stage 2 sleep. Variables of interest included: 1) TST, the total minutes of sleep from sleep onset to final morning wake-up; 2) REM Time (RT), the total minutes of REM sleep from sleep onset to wake-up; 3) REM Percent, the amount of time spent in REM sleep relative to the total amount of sleep time; 4) REM Efficiency, the percentage calculated by dividing total night REM Time in completed REMPs by the duration of all completed REMPs and multiplying by 100; 5) SWS, the sum of minutes spent in Stages 3 and 4 sleep; and 6) WASO, the total minutes awake after sleep onset.

### Statistical analysis

Due to the non-normal distribution of data (verified using the Shapiro-Wilk normality test), non-parametric statistical methods were used to ensure robust and accurate comparisons. Specifically, two-tailed Mann-Whitney U-tests were used to analyze independent data, such as comparing RD1, Total Night RD, and RL between individuals with TRD and HVs at baseline. The Wilcoxon signed rank test was used for dependent data comparisons, including evaluating RD1, Total Night RD, and RL within the TRD group and comparing baseline to post-ketamine treatment. The effect of ketamine on the additional nighttime sleep variables was also evaluated using the Wilcoxon signed-rank test.

In order to investigate the predictive utility of RD1 in distinguishing ketamine responders (defined as those achieving a 50% reduction in HAM-D score one day post-ketamine) versus non-responders, 1000 iterations of hold-out validation were conducted using the Support Vector Machine (SVM) [41] algorithm with a radial basis function. A 70–30 hold-out split (70% for training, 30% for testing) was used, with stratified sampling applied during each iteration to preserve the proportion of responders and non-responders across both training and testing subsets. The receiver operating characteristic (ROC) curve was generated to visualize the performance of the predictive model. In the ROC curve, sensitivity (true positive rate) was plotted on the X-axis against one minus specificity (false positive rate) on the Y-axis. The area under the ROC curve (AUC) was calculated to quantify the predictive performance of the RD1-based model in distinguishing between responders (*n* = 20) and non-responders (*n* = 41) to ketamine treatment among the TRD participants.

All analyses were conducted in MATLAB R2023a (Mathworks, Natick, MA, USA) and SPSS 29. All significant results were checked with Bonferroni-Holm corrections.

## Results

### Baseline total night RD, RD1, and RL in TRD and HV participants

**Fig. 2:**
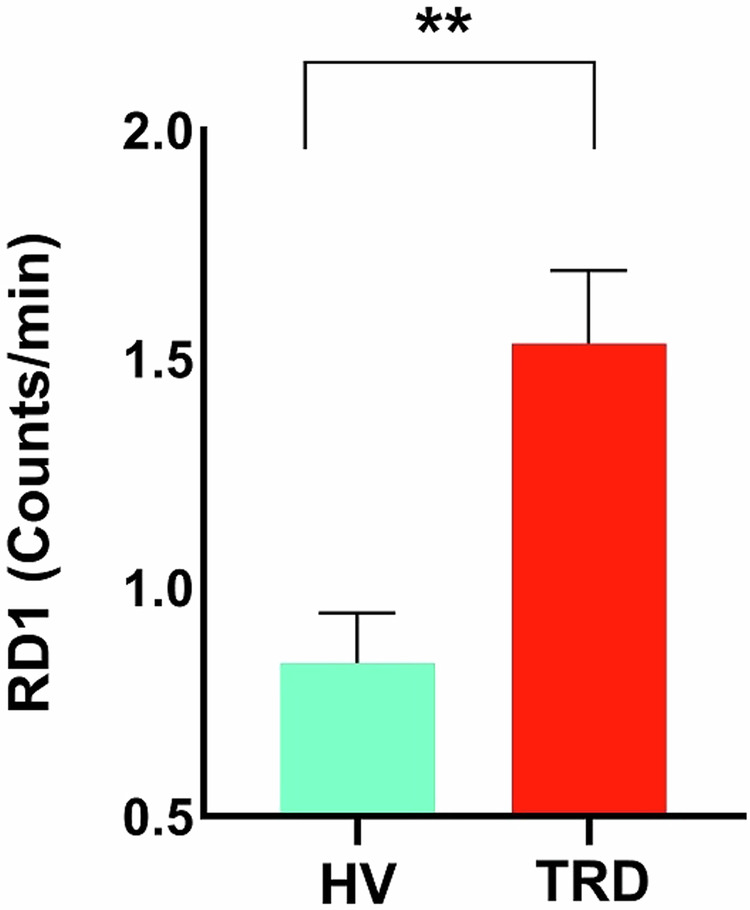
Baseline REM Density in the first REM period (RD1) in individuals with treatment-resistant depression (TRD) versus healthy volunteers (HVs). Bar graphs depicting RD1 density in HVs (green) and individuals with TRD (red). The bars represent the mean RD1 values in each group, and the error bars show the standard error of the mean (SEM). Asterisks indicate statistically significant differences between the groups, as determined by the Mann-Whitney U-test. RD1 was higher in TRD participants than in HVs.

At baseline, RL was shorter in TRD participants than in HVs, while RD1 was higher in TRD participants than in HVs (Fig. 2). No baseline difference in Total Night RD was observed between the two groups (Table 1). Comparisons of additional nighttime sleep variables between individuals with TRD and HVs at baseline are provided in Supplementary Table S1.

**Table 1:** RL, total night RD, and RD1 baseline comparison in TRD vs HV participants.

Sleep Variables	TRD Baseline	HV Baseline	*P*-Value
**RL (min)**	69.8 ( ± 5.7)*	83.8 ( ± 5.6)	**0.01**
**RD1 (Counts/min)**	1.6 ( ± 0.2)	0.8 ( ± 0.1)	**0.002**
**Total night RD (Counts/min)**	1.3 ( ± 0.6)	1.1 ( ± 0.5)	0.1

^*^Mean ± SEM.

Individuals with TRD exhibited higher RD1 and lower RL compared to HVs, as determined by the Mann-Whitney U-test. Adjusted significance levels were verified using the Bonferroni-Holm method, with significant results highlighted in bold. *TRD* treatment-resistant depression, *HVs* healthy volunteers, *SEM* Standard Error of the Mean, *RL* REM Latency, *RD* REM Density, *RD1* REM Density in the first REM period.

### Ketamine’s effects on total night RD, RD1, and RL

**Fig. 3:**
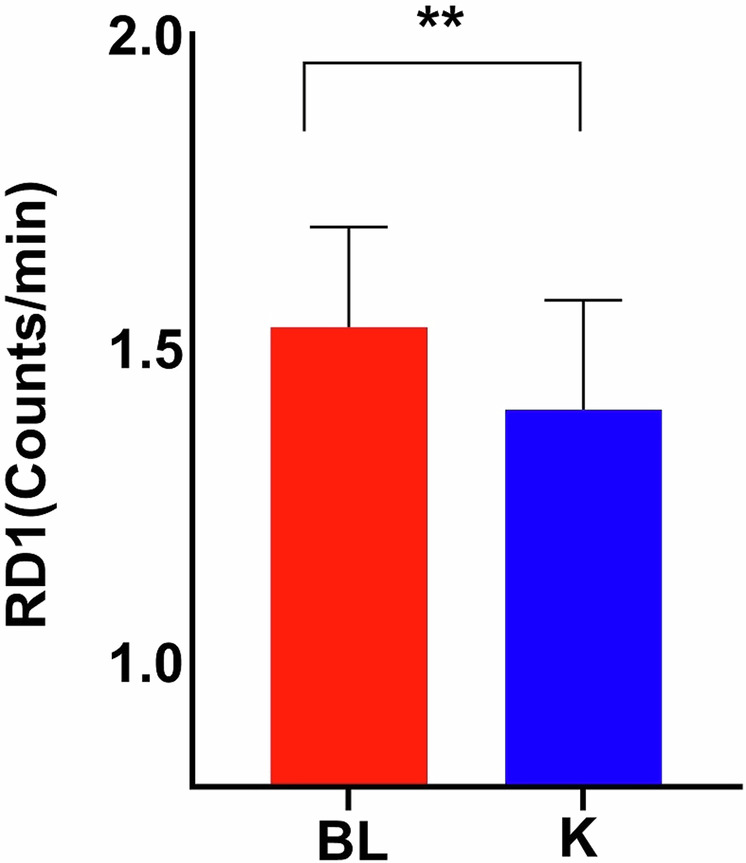
A comparison of REM Density during the first REM period (RD1) at baseline versus post-ketamine in individuals with treatment-resistant depression (TRD). Bar graphs depict RD1 at baseline (red) versus post-ketamine (blue). The bars represent the mean values for RD1 in each group, and the error bars show the standard error of the mean (SEM). RD1 decreased after ketamine treatment in TRD individuals. Asterisks indicate statistically significant differences between the groups, as determined by the Wilcoxon signed rank test. BL baseline.

Ketamine decreased RD1 in TRD participants (Wilcoxon signed-rank Z = 2.69, *p* = 0.007) (Fig. 3). Ketamine had no significant effects on Total Night RD or RL.

### Baseline RD1 in ketamine responders vs non-responders

**Fig. 4:**
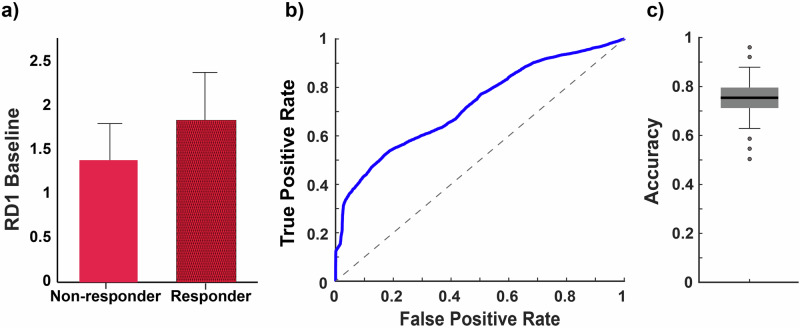
REM Density in the first REM period (RD1) as a predictor of antidepressant response to ketamine. **a** Bar graph representation of RD1 at baseline in individuals with treatment-resistant depression (TRD) who responded to ketamine treatment, defined as a 50% reduction in Hamilton Rating Scale for Depression (HAM-D) score one day post-ketamine, compared to non-responders. The bars represent the mean values for RD1 in each group, and the error bars show the standard error of the mean (SEM). RD1 levels were higher at baseline for responders compared to non-responders. **b** The receiver operating characteristic (ROC) curve generated from 1000 iterations of hold-out validation using the Support Vector Machine (SVM) algorithm to predict responders versus non-responders based on RD1 data. The achieved area under the curve (AUC) was 0.73. **c** Boxplot representation of the accuracy range. Boxplots are given with median, Q1, and Q3 box margins and whiskers indicating 95% confidence intervals, and outliers are indicated by dots. The median accuracy achieved was 0.75, with a 95% confidence interval ranging from 0.63 to 0.88.

Because ketamine reduced RD1 but had no effect on Total Night RD or RL in TRD participants, RD1 was investigated to determine whether it could predict antidepressant response to ketamine. Baseline RD1 levels were compared between ketamine responders (*n* = 20) and non-responders (*n* = 41). Individuals with an antidepressant response to ketamine had significantly higher baseline RD1 levels than non-responders (Wilcoxon signed-rank Z = 1.99, *p* = 0.046; Fig. 4a). SVM evaluation of the predictive potential of baseline RD1 levels for ketamine response in TRD individuals yielded a median accuracy of 0.75 (95%- CI [0.63; 0.88]) and an AUC of 0.73. The mean true positive rate was 65.24, and the mean false positive rate was 40.36. The ROC curve (Fig. 4b) and boxplot of the accuracy range (Fig. 4c) suggest that RD1 may be a favorable predictor of response to ketamine treatment in individuals with TRD.

### Ketamine’s effects on additional nighttime sleep variables

Ketamine increased TST, REM Percent, and REM Time but had no effect on REM Efficiency, RL, SE, SWS, or WASO (Table 2).

**Table 2:** The effects of ketamine on sleep variables in TRD.

Sleep Variables	TRD Baseline	TRD Ketamine	*P*-Value
**TST (min)**	383.4 ( ± 10.2)*	406.8 ( ± 6.78)	**0.008**
**SWS (min)**	25.11 ( ± 4.62)	26.48 ( ± 3.7)	0.1
**WASO (min)**	36.29 ( ± 4.59)	35.49 ( ± 5.07)	0.8
**REM Time (min)**	89.7 ( ± 4.2)	103 ( ± 4.2)	**0.0007**
**REM Percent (%)**	24.8 ( ± 1.5)	26.6( ± 0.99)	**0.006**
**REM Efficiency (%)**	93.2 ( ± 0.66)	98.6 ( ± 0.56)	0.4
**RL (min)**	69.8 ( ± 5.7)	84 ( ± 5.6)	0.06
**SE (%)**	85.02 ( ± 1.9)	88.46 ( ± 1.3)	0.03

^*^Mean ± SEM.

Ketamine increased REM Time, TST, and REM Percent in individuals with TRD, as determined by the Wilcoxon signed-rank test. Adjusted significance levels were verified using the Bonferroni-Holm method, with significant results highlighted in bold. *TRD* treatment-resistant depression, *SEM* Standard Error of the Mean, *TST* Total Sleep Time, *SWS* Slow-wave Sleep, *WASO* Wakefulness After Sleep Onset, *RL* REM Latency, *SE* Sleep Efficiency.

## Discussion

This study examined Total Night RD, RD1, and RL—three key biological markers of depression—with a particular focus on RD1’s potential ability to predict antidepressant response. At baseline, individuals with TRD exhibited higher RD1 and shorter RL, with no difference in Total Night RD compared to HVs. Ketamine reduced RD1 in TRD participants but did not affect Total Night RD or RL. Furthermore, at baseline, RD1 was higher in individuals with TRD who responded to ketamine than those who did not. Use of the baseline RD1 generated a 0.73 AUC and median accuracy of 0.75 (95%- CI [0.63; 0.88]) in predicting ketamine responders versus non-responders. Ketamine also increased some nighttime sleep variables: TST, REM Percent, and REM Time. To our knowledge, this study is the first to investigate RD1 as a predictor of response to ketamine treatment in individuals with TRD. Collectively, the findings underscore the potential usefulness of RD1 for identifying individuals likely to benefit from ketamine, enabling more targeted and effective therapeutic strategies.

At baseline, individuals with TRD had shorter RL, consistent with disturbed REM sleep in depression [10, 16, 42]. Notably, the shortened RL in TRD is consistent with findings in MDD, where RL is considered a trait marker. This may indicate a deficit in the homeostatic mechanisms governing SWS, particularly within the first 90 min after sleep onset, a feature also associated with severity of illness and potential predisposition to depression [24, 43–45]. Surprisingly, however, no differences were found in Total Night RD between TRD and HV participants, which differs from prior findings in MDD [26, 27, 46]. Whether differences in RD are a distinguishing feature between MDD and TRD will require a direct comparison between the two groups. RD typically increases later in the night due to a circadian drive for wakefulness and heightened brain activity [47]. In our participants, however, RD was elevated early in the night, which may be linked to increased arousal [48], an altered circadian drive, or other contributing factors. These potential influences were not examined in this study.

RD1 is considered by some as a trait marker representing an endophenotype of depression [9]. In the current study, treatment with the novel antidepressant ketamine reduced RD1 compared to baseline in individuals with TRD, suggesting that RD1 is not a trait feature in TRD. The effect of ketamine on RD1 was comparable to the reduction in RD1 observed post-ECT [13], underscoring that both ECT and ketamine similarly affect RD1. Interestingly, ketamine had no effect on Total Night RD or RL, indicating its specific impact on RD1 during early sleep in TRD participants. Increased RD1 in depression is likely to involve complex neurobiological mechanisms, serving as an indicator of susceptibility to depressed mood in TRD.

The most salient finding was that elevated baseline RD1 appears to be a marker for antidepressant response to ketamine in TRD (predicted response AUC = 0.73 and median accuracy = 0.75). Two previous studies explored RD as a predictor of treatment response, but neither study examined individuals with TRD or administered ketamine. In individuals with MDD, Göder and colleagues [13] found that higher RD1 levels at baseline predicted better response to ECT, while Lechinger and colleagues [12] found that higher RD at baseline predicted better response to SSRIs and SNRIs. In contrast, Thase and colleagues [49] found that higher RD predicted poorer response to either cognitive behavior therapy or interpersonal psychotherapy in individuals with MDD.

Ketamine’s antidepressant effects in individuals with TRD are thought to be related to sleep homeostasis [19, 50]. For instance, a previous study found that ketamine has early night effects, increasing early-night slow wave activity and improving sleep and mood [19]. This early-night sleep effect is consistent with ketamine’s reduction of RD1 in TRD participants observed in the present study. When ketamine’s effects on additional night sleep variables were investigated, it was found to increase REM Time in TRD participants, a result previously reported by our laboratory [19], suggesting that the present study extended this finding to a larger sample. ECT [13] and atypical antidepressants such as nefazodone and bupropion have similarly been shown to enhance REM Time in MDD [51, 52]; in contrast, other antidepressants such as tricyclic antidepressants (TCAs), monoamine oxidase inhibitors (MAOIs), selective 5-hydroxytryptamine (5-HT), and SSRIs have been shown to decrease REM sleep as part of their antidepressant effects [53]. In the present study, ketamine also increased REM Percent, suggesting that the increase in REM Time was a result of ketamine’s effects on REM sleep rather than merely a consequence of the overall increase in TST observed post-ketamine administration. Furthermore, ketamine had no effect on RL, REM Efficiency, or any of the other investigated variables (WASO, SWS, etc). Ketamine’s effects on RL bear particular mention, given that ketamine, unlike traditional antidepressants that target serotonin, norepinephrine, or monoamine oxidase pathways, did not prolong RL even though conventional antidepressants often increase RL as part of their therapeutic mechanism. In this context, ketamine joins a select group of effective pharmacotherapies that similarly do not increase RL, including agomelatine (a melatonin M1/M2 receptor agonist and 5-HT2C receptor antagonist) [54], mirtazapine, and trazodone (both of which are 5-HT2A receptor antagonists with antihistaminergic properties) [55]. In contrast, sedative tricyclic antidepressants (TCAs) like amitriptyline have been shown to increase RL, with this increase correlating with clinical improvement in depression. However, given ketamine’s acute effects, it remains uncertain whether repeated doses or extended treatment duration might eventually result in an increase in RL. Further investigation is needed to clarify this potential long-term effect.

While several intriguing results emerged from this study, some limitations should also be noted. First, the HV group was smaller (*n* = 41 vs 63) and younger (mean age = 34.49 vs 40.76 years old) than the TRD group. Second, the number of ketamine responders was relatively small (*n* = 20) compared to non-responders (*n* = 41). Third, the study only examined the effects of a single dose of intravenous ketamine, limiting conclusions about repeated or long-term treatment effects. Fourth, while use of the STOP-Bang questionnaire and the SSS likely screened out participants with severe cases of OSA, it may not have excluded those with milder forms of OSA. Finally, the study did not include a group of non-TRD MDD participants, so sleep variables could not be compared between the groups.

In summary, this study found that individuals with TRD exhibited abnormal REM sleep, characterized by increased RD1 and shortened RL compared to HVs. Ketamine treatment improved REM sleep by reducing RD1 as well as increasing REM Time, REM Percent, and TST. Baseline RD1 levels predicted antidepressant response to ketamine, highlighting its potential as a predictor of rapid antidepressant effects in TRD. These findings emphasize the utility of RD1 in identifying those individuals most likely to benefit from ketamine treatment, enabling more targeted and effective therapeutic strategies.

## Supplementary information

Supplemental material

## Data Availability

The data that support the findings of this study are available from the corresponding author upon request.
