# Supplementary material for: REM density predicts rapid antidepressant response to ketamine in individuals with treatment-resistant depression
Source: Neuropsychopharmacology. 2025 Feb 15;50(6):941–6. doi: 10.1038/s41386-025-02066-7 (PMC12032024; doi:10.1038/s41386-025-02066-7)
Supplement: Supplementary file 1 — Supplemental material [file 41386_2025_2066_MOESM1_ESM.docx]

Supplementary Figure S1. CONSORT Diagram for the REM Sleep Study

348 valid nights

71 baseline and 110 post-infusion nights excluded due to protocols that did not meet inclusion criteria

175 baseline nights

173 post-infusion nights

41 nights from HVs

126 nights from individuals with TRD (only from two specific protocols: KMOA* and BP^¥^)

126 nights (63 baseline and 63 post-infusion) from 63 TRD participants (KMOA*= 38, BP^¥^=25)

All 41 nights from 41 HVs (KMOA=41)

Total of 41 HVs

Total of 63 TRD participants

*Ketamine mechanism of action study (KMOA, NCT00088699)

^¥^ Bipolar study (BP, NCT01204918)

TRD: treatment-resistant depression: HV: healthy volunteers


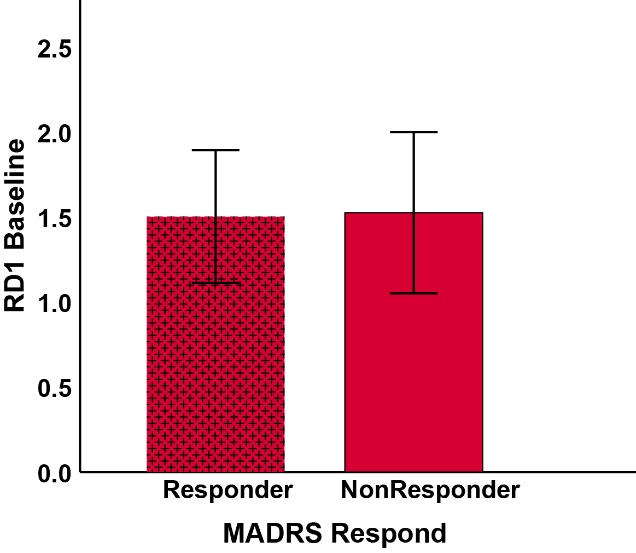


**Figure S2**. Bar graph representation of REM density in the first REM period (RD1) at baseline in individuals with treatment-resistant depression (TRD) who responded to ketamine treatment, defined as a >50% reduction in Montgomery-Asberg Depression Rating Scale (MADRS) score post-ketamine, compared to non-responders. The bars represent the mean values for RD1 in each group, and the error bars show the standard error of the mean (SEM). Baseline RD1 levels did not differ significantly between responders and non-responders.


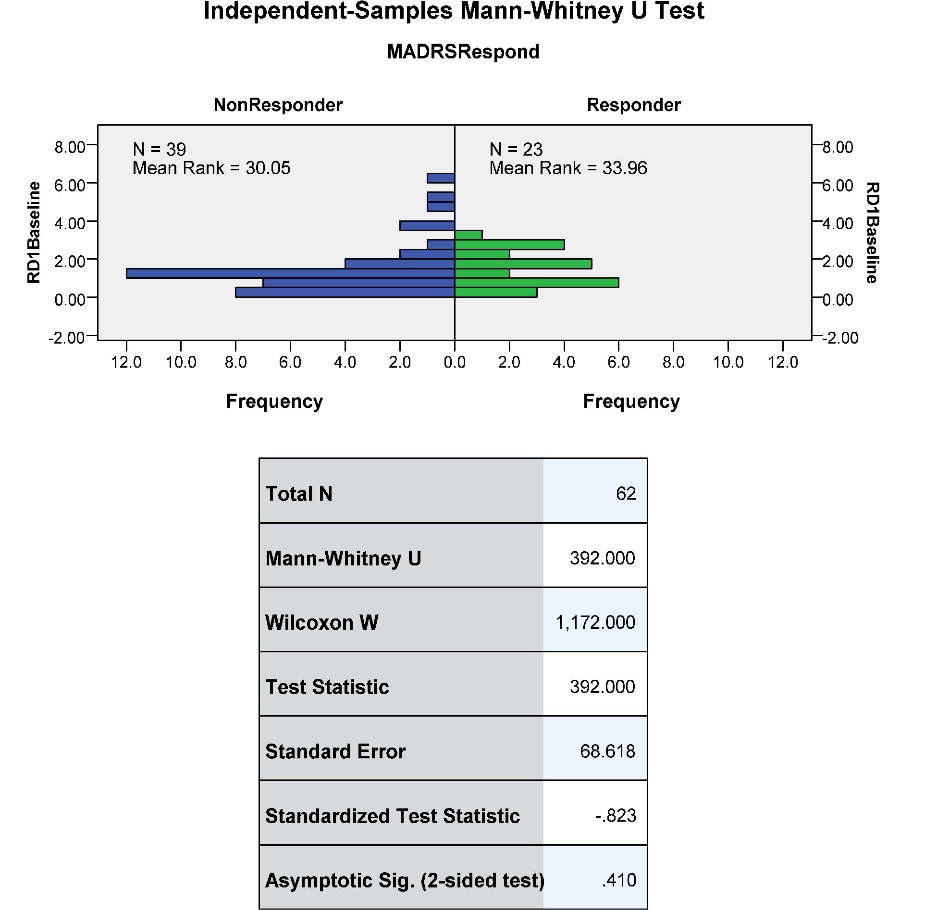


**Figure S3.** Baseline REM density in the first REM period (RD1) showed no significant difference between responders and non-responders, as defined by Montgomery-Asberg Depression Rating Scale (MADRS) scores, based on Independent Samples Mann-Whitney U test analysis.

**Table S1. Baseline comparison of additional night sleep variables between TRD and HV participants**

| Sleep Variables | TRD Baseline | HV Baseline | P-Value |
| --- | --- | --- | --- |
| **TST (min)** | 383.4 (±10.2)* | 411 (± 6.9) | **0.0007** |
| **SWS (min)** | 25.11 (±4.62) | 26 (± 3.5) | 0.2 |
| **WASO (min)** | 36.29 (± 4.59) | 31 (±5.5) | 0.3 |
| **REM Time (min)** | 89 (± 4.2) | 87.9 (± 3.6) | 0.40 |
| **REM Efficiency (%)** | 93.2 (± 0.66) | 95.9 (± 0.56) | **0.005** |
| **SE (%)** | 85.02 (± 1.9) | 88(± 2.6) | 0.07 |

*** Mean** ±SEM

TST and REM Efficiency were significantly lower in individuals with TRD compared to HVs at baseline. However, no other significant differences were observed in nighttime sleep variables between individuals with TRD compared to HVs at baseline. TRD: treatment-resistant depression; HV: healthy volunteer; SEM: Standard Error of the Mean; TST: total sleep time; SWS: slow-wave sleep; WASO: wakefulness after sleep onset; SE: sleep efficiency.
